# Learner Milestones to Guide Decolonial Global Health Education

**DOI:** 10.5334/aogh.3866

**Published:** 2022-11-04

**Authors:** Leah Ratner, Shela Sridhar, Samantha L. Rosman, Jean Junior, Lydia Gyan-Kesse, Jeffrey Edwards, Christiana M. Russ

**Affiliations:** 1 Division of Respiratory Medicine, Department of Pediatrics, Boston Children’s Hospital, Boston, MA, US; 2 Division of General Internal Medicine and Primary Care, Brigham and Women’s Hospital, Boston, MA, US; 3 Global Health Program, Boston Children’s Hospital, Boston, MA, US; 4 Division of Emergency Medicine, Department of Pediatrics, Boston Children’s Hospital, Boston, MA, US; 5 Directorate of Child Health, Komfo Anokye Teaching Hospital, Kumasi, Ghana; 6 Division of Medical Critical Care, Department of Pediatrics, Boston Children’s Hospital, Boston, MA, US

**Keywords:** Decoloniality, Medical Education, Social Medicine, Learning Milestones

## Abstract

The current movement to ‘decolonize’ global health aims to both dismantle colonial frameworks that perpetuate inequity and racism, as well as to rebuild and uplift structures and systems that celebrate indigeneity. However, it is critical to recognize that teaching decoloniality within global health education is more than just the acknowledgement that there are key paradigms missing from current global health education. It is imperative to have a methodology to hold ourselves and our learners accountable to progress in practices and ideals that promote equity-based praxis. In this paper, we propose the creation of a tool to assess learner levels and their progression over time in both recognizing the impacts of colonialism and acting to transform their own global health praxis towards equity and decoloniality.

We developed a model to illustrate an increasing scope and impact of decolonial and global health equity praxis. We hypothesize through this model that the way in which learners engage with power dynamics and structural advocacy at each level is essential to describing learner stages. Based on extensive literature review, existing curricular frameworks, global partner discussion(s), feedback on our pilot curriculum, and adaptation of philosophical theory, these learner milestones were conceptualized. We discuss the inherent challenges in assessment of the complex mix of knowledge, attitude and skills described in these milestones with the understanding that any such assessment would always be formative, as we all continue learning how to do better. We hope these milestones can be utilized to promote critical transformational change in the field of global health. This requires deep self-reflection and examination of existing structures of oppression followed by intentional reparative actions to embody decoloniality in our praxis and advocacy and reimagine global health based on equity and local leadership.

The field of global health has deep colonial roots [1]. It began with the provision of medical care to colonizers afflicted with equatorial diseases. Simultaneously religious institutions sent missionaries to colonies to provide healthcare as a way of introducing ‘Western’ practices and ‘civilizing’ the local population and moving them away from traditional healers and other traditional healing practices [2]. Such colonial frameworks are still omnipresent in the field of global health and can be seen in unequal power dynamics at every level of the practice, including in funding allocation, ‘knowledge production’, and nuance in language.

The current movement to ‘decolonize’ global health aims to both dismantle colonial frameworks that perpetuate inequity and racism, as well as to rebuild and uplift structures and systems that celebrate indigeneity. In this paper, colonialism is conceptualized as ‘the practice of extending and maintaining a nation’s political and economic control over another people or area.’ [3,4]. In order to transform this theory into practice, or develop a praxis, it is vital to integrate decoloniality into global health education in countries who were, and continue to be, colonizing powers. Decoloniality is defined in this paper as the act of participating in this aforementioned practice, though the author group also recognizes there is no one agreed upon definition of decoloniality. The goal of decoloniality education therefore is to reshape global health from its current form, developed to facilitate European colonization and maintain control and oppression, into one of equity, respect, and true partnership globally.

Recent literature has revealed the lack of active and authentic anti-oppressionist education in global health education across institutions [12]. As noted by a recent systematic review on global health education, ‘While the topic of cultural competency/sensitivity is important for enhancing the knowledge, skills, and attitudes of students, these are only one part of the whole patient and system of health care [13].’

Teaching decoloniality requires more than just the acknowledgement that there are key paradigms missing from current global health education. There must be a methodology to hold ourselves and our learners accountable to the goal that learners progress in awareness of structural oppression and in practices that promote equity-based praxis. We propose the creation of a milestones tool to assess learner levels and progression in both recognizing the impacts of colonialism and acting to transform their personal global health praxis and advocacy towards equity and decoloniality. Though there has been a growing movement calling for the decolonization of global health, there still remains a dearth of published tools for assessing learners in this realm [4,5,6,7]. We propose these milestones as a first step in an iterative process to create assessment strategies around equity and decoloniality in global health.

## Existing Curricula and Frameworks

The impetus for these learner milestones arose from the work of three of us (LR, SS, SLR) developing a justice-based, interprofessional, fellow-level decoloniality in global health curriculum in a large academic teaching hospital in the United States. This curriculum, which will be described in a separate publication, targets learners dedicated to a career in global health. As this work evolved, we utilized critical scholastic, interpersonal, and professional feedback from learners and educators in the US as well as in LMIC settings to assess the progress of fellows’ development. However, we lacked a formal method of tracking progression over time and attainment of critical milestones. After review of several previously published frameworks of learner progression, we developed a milestones metric to integrate with our curriculum.

Our work was modeled off several existing rubrics including the Fair Trade Learning Rubric [5], the VALUE rubric [8], an asset based model on community involvement [7] and Arnstein’s ladder of citizen participation [6]. Additionally work by Harvey et al. [9] identifies five main categories in which learners should be competent to address structural education in practice. They are: (1) Describe the role of social structures in producing and maintaining health inequities globally, (2) Identify the ways that structural inequalities are naturalized within the field of global health, (3) Discuss the impact of structures on the practice of global health, (4) Recognise structural interventions for addressing global health inequities, and (5) Apply the concept of structural humility in the context of global health [9].

Additionally, a literature review of existing global health competencies helped inform our learner milestones. Steeb et al. [10] developed entrustable professional activities in global health that describe critical skills that global health trainees should attain [10]. Similarly, the Consortium of Universities for Global Health has defined learner levels ranging from Level 1: Global Citizen to Level 4: Advanced level (those with a masters or doctorate in global health subject matter) [11]. A scoping review of global health competencies by Schlieff et al. [12] makes the powerful conclusion that these competencies must respond to learner needs and career trajectories [12]. Though there are many established global health competencies in the literature, they are often very broad and difficult to use to track the progress of individual learners. Furthermore, while these competencies may consider issues of equity and social justice, most leave out issues of colonialism and racism in global health competency frameworks [14].

## Relevant Philosophical and Ethical Theories

In clinical medicine, traditional learner levels are determined by years of clinical training, regardless of one’s non-clinical experience. However, in the pursuit of global health equity, the level of formal clinical training or years of global health experience may be incongruent with ‘expertise’. Rather, other factors may be more relevant including lived experience, other coursework, participation in political advocacy efforts, self-reflection, and the ability to question (Eurocentric) epistemological frameworks, or how one has come to ‘know what we know.’ True decolonial praxis as said by Thirusha Naidu ‘begins with an initial realization or awareness of one’s position within the colonial matrix of power followed by the reflecting or deliberation, or a grappling with real-life struggles that are encountered in confronting the oppressive operations of the colonial matrix of power [15].’ Even the most advanced clinicians with years of global health experience may be novice learners of decoloniality. Our proposed learner milestones aim to help learners and educators think critically about the power and knowledge structures on which much of global health practice is based, and thereby foster growth and progress in decolonial praxis.

Our milestones also build off of literature on anti-racism, specifically Kendi’s depiction of learning about antiracism that involves moving away from a zone of fear towards a zone of growth [16]. Our milestones show similar progression, with increasing levels of depth and insight [16,17]. Though the work of anti-racism domestically draws many parallels to anti-coloniality, we recognize there are many unique historical and current issues that do not overlap, thus further motivating the creation of specific decoloniality learner milestones.

## Milestones Development

**Figure 1:**
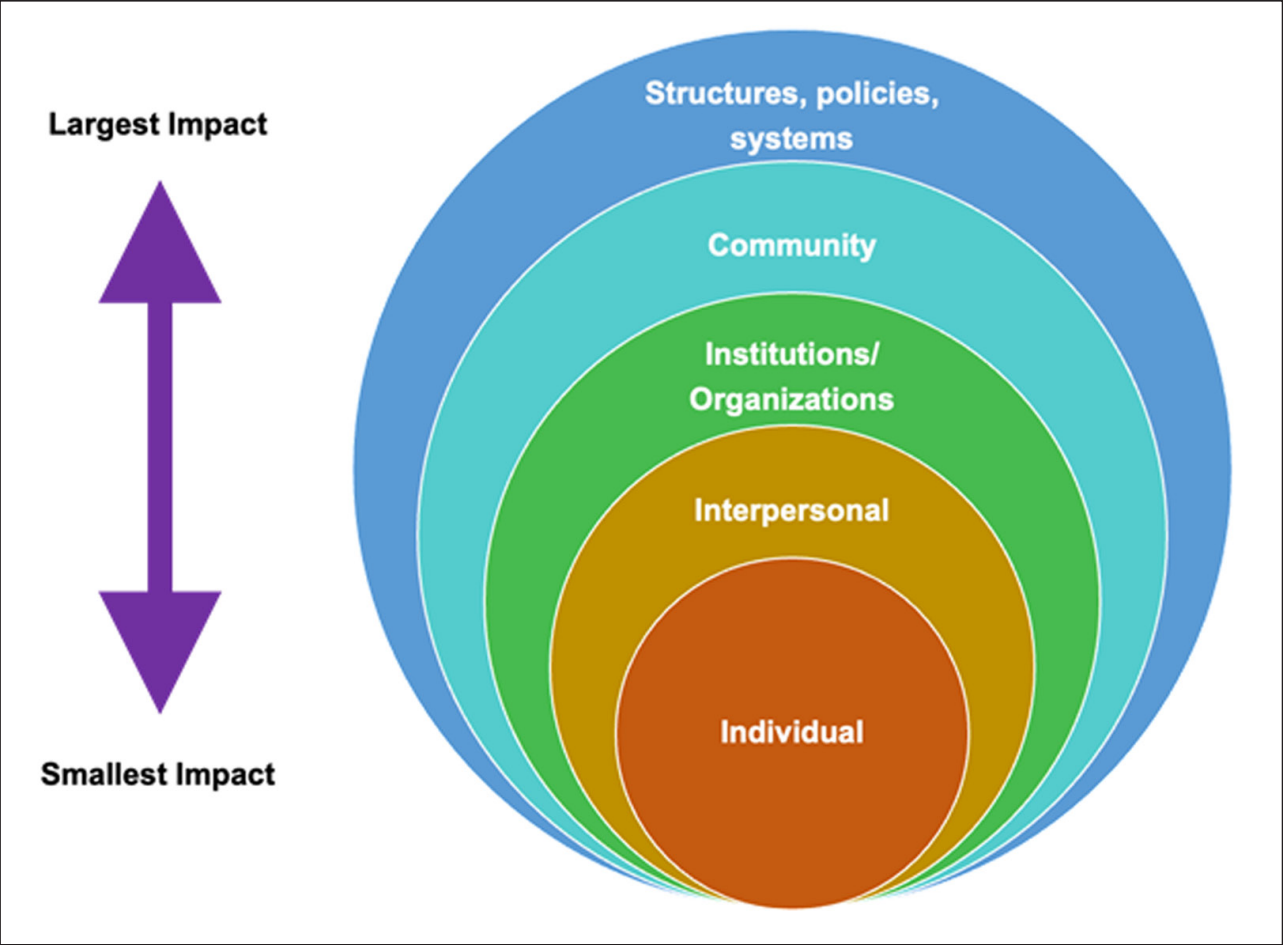
Adapted Social ecological model for levels of learner progression [18].

We adapted a social ecological model (see Figure 1) [18,19,20], to illustrate an increasing scope and impact of decolonial and global health equity praxis, extending outwards from self (individual), to relationships (interpersonal), through institutions, community, and finally structures, policies, and systems. These milestones were developed to measure how learners from historically and ongoing colonizing countries (typically high-income countries) engage with power dynamics and structural advocacy at each level of this model. Our milestones also emphasize the importance of identifying discrepancies between voiced intent and actual practice. Incorporating the above principles, followed by iterative discussions with and feedback from global partners from historically and ongoing colonized settings (typically low- and middle-income countries) we developed the learner milestones below, with concentric scopes of impact as the learner progresses.

Learning milestones:

I. **Pre-Contemplative Learner** – A learner in this stage may consider themselves ‘color blind’ and assume racial and socioeconomic differences are unimportant or irrelevant. They may not have had much interpersonal experience with people who have different identities than their own. They may have little to no recognition of the ongoing impact of colonial histories or white privilege. They may consider that all global health work is ‘noble’ and effective at helping ‘help those less fortunate.’
  Power Dynamics: This learner may knowingly or unknowingly assume they are superior to those from resource-denied settings. They often fail to see that the ways in which they communicate with global partners are disempowering, or fail to value partners’ input, knowledge, or skills.
  Structural advocacy: This learner has not reflected on how large-scale historical, socioeconomic, or political forces (e.g., colonialism, racism, the distribution of financial and political power) have created injustices that persist in today’s world, or in what ways they derive privilege from these injustices.
II. **Contemplative Reflective Learner** – A learner in this stage is newly embracing decolonial concepts. They are learning to identify and name their own biases at an individual level.
  Power Dynamics: This learner recognizes the existence of problematic colonial history and how resultant colonial supremacy persists in power and positionality in global health today. They are starting to learn and practice more equitable interpersonal interaction. They may not yet know how to move beyond articulating concepts of decoloniality to meaningfully prioritizing local viewpoints and equitably collaborating with global partners.
  Structural Advocacy: This learner may recognize the importance of large-scale historical, socioeconomic, and political forces on themselves and their partners, but does not yet know how to take meaningful action to counter these forces in their global health work.
III. **Critical Action Learner** – A learner in this stage can use their individual reflection to effectively collaborate at an interpersonal level. They are able to take action at the individual, institutional, or community level to actively work to change inequities.
  Power Dynamics: This learner prioritizes partner’s goals in resource-constrained settings, leadership and decision making while decentering their own role.
  Structural Advocacy: This learner recognizes how inequities are shaped and perpetuated by historical, socioeconomic, and political forces and takes meaningful action to advocate for local structural changes to promote equity and decoloniality through their own work does not yet demonstrate the ability to call in others to similar meaningful actions or work at a broader policy or systems level for change.
IV. **Transformative Action Learner** – A learner in this stage effectively acts to dismantle racist and colonial policies at a systems level. This is an aspirational level.
  Power Dynamics: This learner inspires high-income country (HIC) stakeholders to transform global health practice by placing power and money in the hands of partners from historically and ongoing colonized settings.
  Structural Advocacy: This learner works to dismantle global structural forces which are perpetuating inequities and the colonial matrix of power by calling in those around them to join in this work. They work to decenter the role of HIC stakeholders and to allow their global partners to reinvent the field as one based on respectful partnerships and in which local partners hold the leadership role in priority setting, funding, and project management.

## Discussion

It is critical that we deconstruct the colonial matrix of power on which global health is based and transform it into a field based on principles of social justice, ethics, and equity [20,21]. To achieve this goal, we must dismantle existing power asymmetries and transform individual and community practice and structures of oppression. Global health education leaders must highlight educational competencies that reflect this goal in all areas of practice; including but not limited to, global health research [22], governance [23], and partnerships [24].

It is important to acknowledge that ultimately these milestones were designed for and by those educated in systems of power and therefore have inherent bias and blind spots to the lived experiences of those educated within systems of oppression, notably to the continued structures upholding this oppression. Though we actively sought feedback from global partners we also recognize that this burden should not fall on our colleagues from formerly colonized settings to educate learners from colonizer countries. While insight, feedback, and critique from our global colleagues is essential, the work of dismantling current educational paradigms is our responsibility as global health educators with the unearned privileges inherent in being from a colonizing country. This is our work to do and we hope these milestones will support the unlearning and decoloniality praxis necessary for those educated in settings of power aiming to practice and promote global health equity. Similar milestones for those educated in formerly colonized settings and systems of oppression aiming to practice (global) health equity are likely also needed but would be likely quite different and would need to be tailored to that setting and context and be grounded in trauma-informed methodologies.

Our conceptualization of learner milestones is a first step in the development of tools to aid in assessing global health learner progression towards effective praxis in decoloniality and global health equity. However, we recognize that there are numerous challenges in developing a true evidence base for valid, reliable assessments in this complex learning domain. Similar to several of the ACGME competencies, equity and decoloniality praxis in global health is challenging to measure and must be recognized as only ‘achievable’ through lifelong learning [25]. Our learner milestones are thus meant to be formative, not summative [26]. They establish the importance of identifying the learner’s starting point and goals, and allowing learners to recognize their progression across the biopsychosocial model in real-time. The learner milestones serve as a tool for measuring the process of transformation and changes in practice rather than objective knowledge around certain subject matters. The learning, by definition, must be unconventional. Testing or an ‘end-point’ would be antithetical and possibly harmful to learners’ decolonial journey.

We hope these milestones will ultimately be incorporated into assessment tools used at regular intervals by learners themselves, their peers and colleagues (in both resource-rich and resource-denied settings), and global health educators. Feedback from colleagues in formerly and ongoing colonized settings is particularly important to understanding how well learners incorporate decolonial practice into their work in resource-denied contexts. This feedback must be obtained carefully with deep understanding of power imbalances and potential unintended consequences, ideally through robust longitudinal partnerships. Furthermore, while we recognize that self-assessment can be inaccurate, (allowing for both over- or under-estimation of learner abilities) [27] we believe that there is value in the use of milestones for self-assessment, as a way to foster learner reflection on their approach to global health practice and to help guide their plans for future growth. No matter who is doing the assessments, the sensitive nature of these topics demands that care be taken in creating a safe environment for those receiving and giving feedback. Working toward a decolonial global health praxis requires shared vulnerability, progressive self-awareness, and humility.

More work is needed to apply these milestones to specific learning objectives in global health and to develop assessment tools that provide valid, reliable measurements. Additional research is also needed to show whether these milestones are helpful to both learners and educators, and to measure if they impact future learning and practice [25]. The work of developing assessment tools in decoloniality and equity is not easy and requires input from a diverse group of individuals from a wide variety of global health contexts. However, it is critical that we embark on this journey of transforming the way we teach and assess global health education to prioritize equity and decoloniality rather than perpetuating the colonial matrix of power currently inherent in so much of the way global health is conceived, taught and practiced.

## Conclusion

Our learner levels establish the importance of recognizing where the learner has begun, where the learner is headed, and the amount of progress the learner has made. Learners can anticipate pathways of growth and skill development across the biopsychosocial model. Transformational change in the field of global health relies on deep self-reflection and intentional reparative actions to decolonize our praxis and reimagine global health based on equity and local leadership. These learner levels aim to provide learners and educators with a tool to support a transformational change towards equity in the field of global health.
